# Fabricating and Characterizing the Microfluidic Solid Phase Extraction Module Coupling with Integrated ESI Emitters †

**DOI:** 10.3390/mi9050212

**Published:** 2018-05-01

**Authors:** Hangbin Tang, Quan Yu, Xiang Qian, Kai Ni, Xiaohao Wang

**Affiliations:** 1 Division of Advanced Manufacturing, Graduate School at Shenzhen, Tsinghua University, Shenzhen 518055, China; thb15@mails.tsinghua.edu.cn (H.T.); yu.quan@sz.tsinghua.edu.cn (Q.Y.); ni.kai@sz.tsinghua.edu.cn (K.N.); wang.xiaohao@sz.tsinghua.edu.cn (X.W.); 2 The State Key Laboratory of Precision Measurement Technology and Instruments, Tsinghua University, Beijing 100084, China

**Keywords:** microfluidic chip, solid phase extraction, mass spectrometry, two-phase flow focusing, integrated electrospray ionization (ESI) emitter, on-chip solid phase micro-extraction (SPME) module, pretreatment technology

## Abstract

Microfluidic chips coupling with mass spectrometry (MS) will be of great significance to the development of relevant instruments involving chemical and bio-chemical analysis, drug detection, food and environmental applications and so on. In our previous works, we proposed two types of microfluidic electrospray ionization (ESI) chip coupling with MS: the two-phase flow focusing (FF) ESI microfluidic chip and the corner-integrated ESI emitter, respectively. However the pretreatment module integrated with these ESI emitters is still a challenging problem. In this paper, we concentrated on integrating the solid phase micro-extraction (SPME) module with our previous proposed on-chip ESI emitters; the fabrication processes of such SPME module are fully compatible with our previous proposed ESI emitters based on the multi-layer soft lithography. We optimized the structure of the integrated chip and characterized its performance using standard samples. Furthermore, we verified its abilities of salt removal, extraction of multiple analytes and separation through on-chip elution using mimic biological urine spiked with different drugs. The results indicated that our proposed integrated module with ESI emitters is practical and effective for real biological sample pretreatment and MS detection.

## 1. Introduction

The microfluidic chip has the ability to integrate basic operation units in the field of chemistry and biology such as sample preparation, reaction, separation and detection into a single chip with few square centimeters; Thus, the basic characteristics and the significant advantages of such so-called lab-on-chip technology are the flexible combination and scale integration of functional module on the tiny platform [1,2]. As a high-sensitivity technology, electrospray ionization (ESI) mass spectrometry (MS) is the suitable choice for qualitative or quantitative analysis of unknown trace matrix [3,4,5,6,7]. Koster et al. [8] and Feng et al. [9] conducted a thorough summary of the materials and fabrication methods used for microfluidic chips coupling with MS. Among these materials, polydimethylsiloxane (PDMS) was widely used because of its chemical inertness, optical property, and low cost. Moreover, PDMS was the most commonly used polymer material in soft lithography which PDMS microchip could be fabricated in large quantities with accurate mold fabricated using lithography. Stefan Ohla et al. [10] reviewed the developments in chip electrophoresis or chip chromatography to MS field and commented recent trends to face current and future challenges in chip-based biochemical analysis. Chip coupling with MS will be of great significance to the development of relevant instruments involving sample analysis, food and drug detection, biochemical analysis and so on. At present, one of the basic application modes is that the sample is pretreated with a series of extraction, reaction, separation, etc. in the microfluidic chip channel and then electrospray is formed by an integrated nozzle.

In our previous works [11,12,13], we proposed two types of microfluidic electrospray ionization (ESI) chip coupling with MS: the two-phase flow focusing (FF) ESI microfluidic chip and the corner-integrated ESI emitter, respectively. However, those chips were not integrated with any pretreatment modules and the advantages of the microfluidic chips were not fully presented. Among the emerged pretreatment technologies for on-chip integrating, liquid-phase micro-extraction (LPME) [14,15] and solid-phase micro-extraction (SPME) [16,17,18,19,20] are two promising options. LPME in microfluidic chips is used to transfer substances from one phase to another by the solubility or distribution ratio of the two phases which produce stable laminar flow in the microchannel; SPME is a sample preparation technology that a solvent (aqueous or organic) is used for adsorption and elution of the sample. It is based on different distribution coefficient [21] in solid phase and solution. When the solution passes through solid phase extraction particles, the particles adsorb the target analytes in the solution, and the target analytes on the particles also dissolve in the solution, which is a dynamic process. Particles can initially adsorb a large amount of the target analytes in the solution until reaching adsorption saturation, so the distribution coefficient at each stage is different [21]. A lot of research groups have published papers in these areas. Asl et al. [22] introduced an on-chip LPME coupled with high performance liquid chromatography for the analysis of levonorgestrel (Levo), dydrogesterone (Dydo) and medroxyprogesterone (Medo) as the model analytes in biological samples. Gaspar et al. [23] filled silica particles to chromatographic separation chip by the method of non-sintering, realizing extraction and separation of three kinds of antibiotics on chip. Nagy et al. [24] made parallel extraction channel and filled C18 silica particles into bottleneck structure, realizing the rapid high-throughput separation of two kinds of dye. In this paper, we focus on integrating the bottleneck SPME module with our previous proposed on-chip ESI emitters. We proposed new fabrication process, optimized the elution condition and realized multiple analytes separation via such SPME module on chip. Comparing with previous constructive works [25] in which the on-chip micro-SPE module is couple with a capillary ESI emitter to eliminate salts in the complex sample matrix, the improvement of our proposed work is straightforward. Firstly, coupling the SPME module with the integrated ESI emitter is a dead volume free configuration; and secondly, we not only focus on the problem of eliminating salts but also separation of multiple analytes in the complex sample matrix under the best elution conditions. The application prospect of such on-chip SPME module coupling with ESI MS is expected to be widespread, especially in food and medicine analysis in the future.

## 2. Materials and Methods

### 2.1. Materials and Equipment

HPLC-grade methanol and HPLC-grade acetonitrile were purchased from Merck KGaA (Darmstadt, Germany). Formic acid was purchased from Aladdin (Shanghai, China). Reserpine and clenbuterol hydrochloride was purchased from ANPEL (Shanghai, China); mimic urine was purchased from XingHeng Co., Ltd. (Dongguan, China). PDMS elastomer base and curing agent (Sylgard184) were purchased from Dow Corning (Midland, MI, USA). SU-8 photoresist was obtained from Microchem Co. (Naton, MA, USA). MCX particles and C18 particles were purchased from ANPEL Laboratory Technologies (Shanghai) Inc. (Poly-Sery MCX, CNW, Shanghai, China), the diameter of particles were about 30 µm to 40 µm. All liquid and air were supplied to the microfluidic chip through short stainless steel tubes embedded in the reservoirs using a pneumatic pressure controller (MFCS, Fluigent, Paris, France). This pneumatic pressure controller allowed almost non-fluctuating flow, which was essential to form a steady Taylor cone. The high voltage generated by a power supply module (Dongwen High Voltage Power Supply Co., Ltd., Tianjin, China) was applied on the stainless steel tube of the liquid phase. A high-speed camera (ORCA-flash, Hamamatsu, Shizuoka, Japan) mounted on an inverted optical microscope (Eclipse TE 2000-U, Nikon, Tokyo, Japan) was used to observe the experiments. An ion trap mass spectrometer (Thermo Fisher Scientific Inc., Waltham, MA, USA) was coupled to the microfluidic chip, and MS data were collected by the computer.

### 2.2. Microfluidic Chip Design and Fabrication

**Figure 1:**
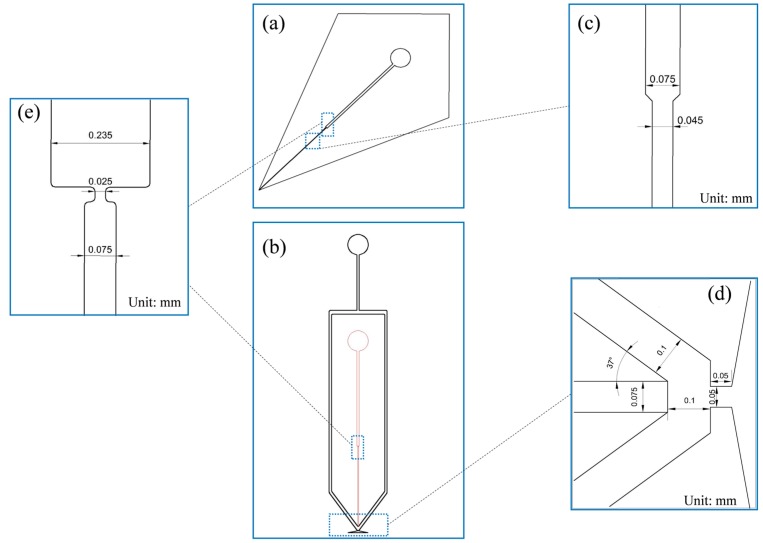
Photo mask, bottleneck structure size and nozzle size of integrated-corner electrospray ionization (ESI) emitter and flow focusing (FF) ESI emitter: (**a**) Photo mask of the integrated-corner ESI emitter; (**b**) Photo mask of the FF ESI emitter; (**c**) Nozzle width of integrated-corner ESI emitter; (**d**) Nozzle width of FF ESI emitter; (**e**) Bottleneck structure size.

As described previously, the main designing consideration is to coupling the SPME module to our microfluidic ESI emitters: the two phase FF ESI microfluidic chip and the corner-integrated ESI emitter, respectively. All the chips were bonded by two PDMS slabs with the same mirror structures, referred as top and bottom slabs. For each chip, the SPME module used a bottleneck structure which is located on the upstream of the ESI emitters to implement the packing; and the size of the bottleneck structure ensured that the particles can be retained. Comparing to other structure for SPME particles retaining, such bottleneck structure is much easy implemented in the PDMS micro-channels using soft lithography. We tested different sizes of bottleneck structure and found that the optimal width ahead bottleneck, in bottleneck and behind bottleneck were 235 µm, 25 µm, 75 µm respectively, as shown in Figure 1e, which ensures filling the SPME particles into the pretreatment channel without leakage or clogging. With the fixed width, the depth and the length of the SPME module determines how many particles we can be filled in to the SPME channel. Considering the cast mold ability and tiny size of the chip, 260 µm was chosen as the depth and 10 mm as the length, respectively. The 260 µm depth SPME channel troubled the downstream ESI nozzles, as we found it in our previous work [11] that the small size of the cross section of the nozzle ensured the best spraying stability (around 40 µm). So four configurations of the two kinds of microfluidic chips were tested in this paper, that is to say, 260 µm depth SPME with 260 µm depth corner-integrated ESI and FF ESI, and with 40 µm depth corner-integrated ESI and FF ESI, respectively. However, the fabrication processes will become a little complicated as for a 40 µm depth nozzle, one more lithography layer should be applied for the nozzle independently comparing to the 260 µm depth nozzle.

Figure 1a shows the photo mask of corner-integrated ESI emitter. Figure 1c shows the enlarged view of the upstream of the bottleneck where the width of the channel was shrink to 45 µm to the nozzle. Figure 1b shows photo mask of two phase FF ESI microfluidic chip. This structure and corresponding fabrication process avoided the trouble of cutting along the edge of the nozzle outlet. The trumpet-shaped outlet was instead cut far from the nozzle, thus greatly improving the craftwork and allowing the rapid mass production of microfluidic chips. This chip has two kind of channels: liquid channel and air channel. The depth of air channels is 520 µm. Other relevant dimensions of nozzle are shown as Figure 1d. All photo masks for lithography (patterns illustrated in Figure 1a,b) were designed by AutoCAD (version 13, Autodesk, Inc., San Rafael, CA, USA) and were manufactured by Qingyi Precision Mask Making Co., Ltd. (Shenzhen, China).

**Figure 2:**
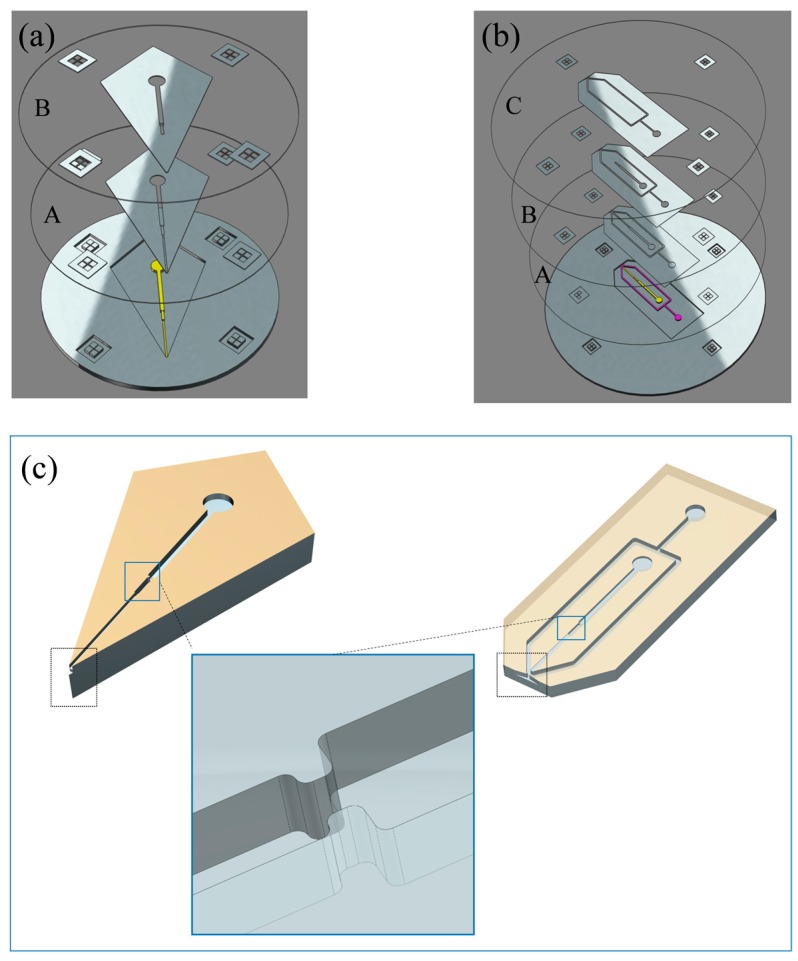
Fabrication process of microfluidic solid-phase micro-extraction (SPME) module ESI emitter: (**a**) Photo mask A, photo mask B and SU-8 master mold of integrated-corner ESI emitter; (**b**) Photo mask A, photo mask B, photo mask C and SU-8 master mold of FF ESI emitter; (**c**) illustration of single polydimethylsiloxane (PDMS) slabs of both chips.

All above mentioned microfluidic chips were fabricated by standard multilayer soft lithography techniques [26]. The fabrication process has been shown in our previous works as in the Supplementary Materials Figure S1 of reference [11], but with some modifications. Briefly, to fabricate microfluidic SPME module with corner integrated ESI emitter, a 3 inches silicon wafer template was treated in oxygen plasma (PDC-M, Chengdu Mingheng Science & Technology Co., Ltd., Chengdu, China) to prevent the photoresist from spalling. The negative photoresist (SU-8 2025) was then poured on the silicon wafer. After spinning with the speed of 3000 rpm for 30 s and soft-baking at 95 °C for 6 min, the photoresist was exposed via photo mask A as shown in Figure 2a, which served as the liquid channel layer, providing an orifice and a channel for the solution. The dosage for UV exposure was about 150~160 mJ/cm^2^. After post-baking which temperature was 95 °C for 6 min and cooling, a second layer of negative photoresist (SU-8 2100) was applied at the top of the liquid channel layer without developing uncross-linked photoresist. After spinning with speed of 2500 rpm for 30 s and soft-baking at 95 °C for 30 min, photo mask B was placed on the second layer photoresist for exposure, which was aligned with the liquid channel layer by a ultraviolet (UV) aligner, also as shown in Figure 2a. The dosage for UV exposure was about 240–260 mJ/cm^2^. This second layer determined the depth of the bottleneck structure for filling in more particles and kept the nozzle depth unchanged. After post-baking which temperature was 95 °C for 6 min and cooling, the SU-8 photoresist layers were developed in propylene glycol methyl ether acetate and were then placed on the thermostatic platform for 2 h for hard-baking. This SU-8 master mold served as the top layer and bottom layer of our micro-channel structure. The final structures of the SU-8 master mold are illustrated in Figure 2c. The depth of the nozzle and bottleneck of top layer or bottom layer SU-8 master mold processed in this way were 20 µm and 130 µm respectively. We also only exposed second layer photoresist using photo mask A without the requirement of aligning to the mask B, so the depth of the nozzle and bottleneck were same, which is to say 130 µm, this configuration ease the fabrication processes but has a larger open nozzle.

The SU-8 master molds were modified with vapor-phase TMCS (chlorotrimethylsilane) to assist the release of PDMS membranes. PDMS base monomer and curing agent were mixed at 10:1 and 5:1 weight ratios and then poured for the top and bottom PMDS slabs, respectively. After degassing under vacuum, these two half-pieces were cured in an oven at 80 °C for 0.5 h. Subsequently two PDMS slabs (the thickness of each PDMS layer was 2 cm) were peeled off from the master molds and the inlet holes were drilled at the top by using a punch (tip diameter of 0.75 mm). Both PDMS slabs, which were treated in oxygen plasma for 2 min (PDC-M, Chengdu Mingheng Science & Technology Co., Ltd., Chengdu, China), were then bonded together by using an xyz-manipulator (Beijing Optical Century Instrument Co., Ltd., Beijing, China). Following assembly, the PDMS microfluidic chip was cured at 80 °C for 72 h to enhance the strength of the bonding and to eliminate the MS background from PDMS.

The fabrication process of microfluidic SPME module integrated with FF ESI emitter is as same as integrated-corner ESI emitter. It had three layer negative photoresists, SU-8 2025, SU-8 2100 and SU-8 2100. The spinning speed of each layer was 3000 rpm, 2500 rpm, 2000 rpm and the dosage for UV exposure was about 150~160 mJ/cm^2^, 240~260 mJ/cm^2^, 240~260 mJ/cm^2^, respectively. The final structures of the SU-8 master mold are illustrated in Figure 2c. The single layer depth of the liquid channel of nozzle, bottleneck structure and air channel in this way were 20 µm, 130 µm and 260 µm, respectively. We also fabricated the chip that the depth of liquid channel of nozzle and bottleneck structure were same, which is to say 130 µm and the air channel was not changed. 

### 2.3. SPME Particles Filling and Expriment Setup

We filled of a 50 mL centrifuge tube with SPME particles (MCX or C18) and deionized water to make particles/water suspension. 80 μL suspension solution was inhaled by pipette and injected into liquid channel. SPME particles entered into the liquid channel with deionized water, in situ polymerization at the bottleneck structure, forming a micro column. Clogging issues are rare in a large number of experiments of SPME on chip we had done. This was related to spherical shape of particles, random in-situ polymerization in the channel, optimizing the bottleneck width, and processing technology. After that, 80 μL methanol was inhaled by pipette and injected into liquid channel to activate particles and remove some impurities on the surface of particles and channel. Finally, deionized water was injected into channel from liquid storage container to remove methanol and the on-chip SPME module was prepared for usage. 

**Figure 3:**
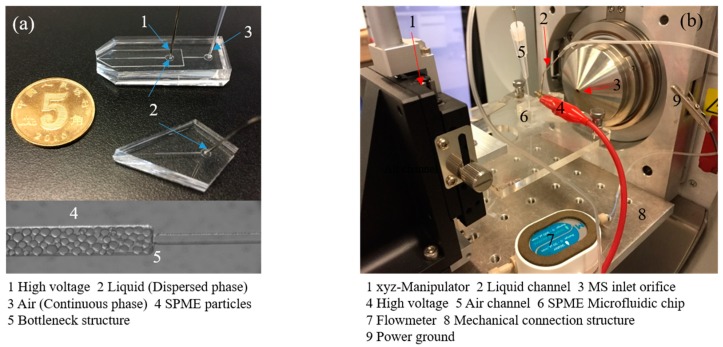
Microfluidic SPME module and laboratory-built experimental platform: (**a**) Microfluidic chips with SPME particles at bottleneck structure and (**b**) The configuration of the laboratory-built experimental platform coupling with mass spectrometry (MS).

The microfluidic chip shown in Figure 3a was produced by the above fabrication and particles filling processes. Figure 3b displays the configuration of the microfluidic chip held by a laboratory-built platform and coupled to the MS. The distance between the chip emitter and the MS inlet orifice is about 2–10 mm, which is adjusted by a xyz-manipulator. For corner-integrated ESI emitter, the pressure of liquid flow channel is 300 mbar. The voltage between the MS inlet orifice and the integrated ESI emitters is 5.5 kV. For FF ESI chip, the pressure of the gas flow channel and liquid flow channel are 900 mbar and 300 mbar respectively. The voltage between the MS inlet orifice and the integrated ESI emitters is 5.5 kV.

### 2.4. SPME Experiments

In order to compare the results of different chips structures mentioned above, we firstly using pure standard samples for SPME experiments. 0.82 µM reserpine dissolved in 1/4 (*v*/*v*) acetonitrile/water was worked as standard sample solution. Organic phase is added to solution in order to having better solubility of reserpine comparing with dissolving in water directly. The content of acetonitrile in the solution is relatively low comparing to the elution conditions, which didn’t affect the adsorption reserpine by SPME particles. In such single analytes experiment, we selected MCX as the SPME particle that is a kind of solid phase extraction particles of mixed cationic exchange with good selectivity to alkaline and neutral compounds. With the difference of silica gel matrix which is traditional SPE particles, MCX matrix had modified styrene-divinyl benzene copolymer which had the hydrophilic and hydrophobic groups on the surface, and it has good water infiltrating. It is very stable within the scope of the pH 0–14, suitable for alkaline and neutral compounds in serum, urine, and food.

The standard reserpine solution was injected into liquid channel from the liquid storage container under pressure driven, through the MCX particles which absorbed and enriched reserpine on the surface unceasingly until adsorption saturation. We respectively set the experiment of sample extraction for 20 min, 30 min, 40 min, 50 min and 60 min to find the saturation point. The results were similar to those of the other groups, except for 20 min. So we thought experiment took 30 min to have researched saturation point. After the sample extraction, we used deionized water to rinse particles. During the elution stage, different acetonitrile and water ratio (50–90%) with 1% formic acid were tested to find the optimal elution conditions. Furthermore, under such optimal elution conditions, we compared the MS signal with above mentioned four chip configurations, namely corner-integrated ESI with 40 µm and 260 µm depth nozzle and FF ESI with 40 µm and 260 µm depth nozzle, respectively.

Furthermore, under optimal elution conditions and optimal chip configurations, mimic urine sample spiked with single or multiple drugs was treated as real bio-chemical sample with drug metabolite. The experiments processes were the same as the standard reserpine solution with only different that for multiple analytes, in order to get a better adsorption effect, we chose C18 SPME particles.

## 3. Results and Discussion

### 3.1. Single Standard Sample SPME

**Figure 4:**
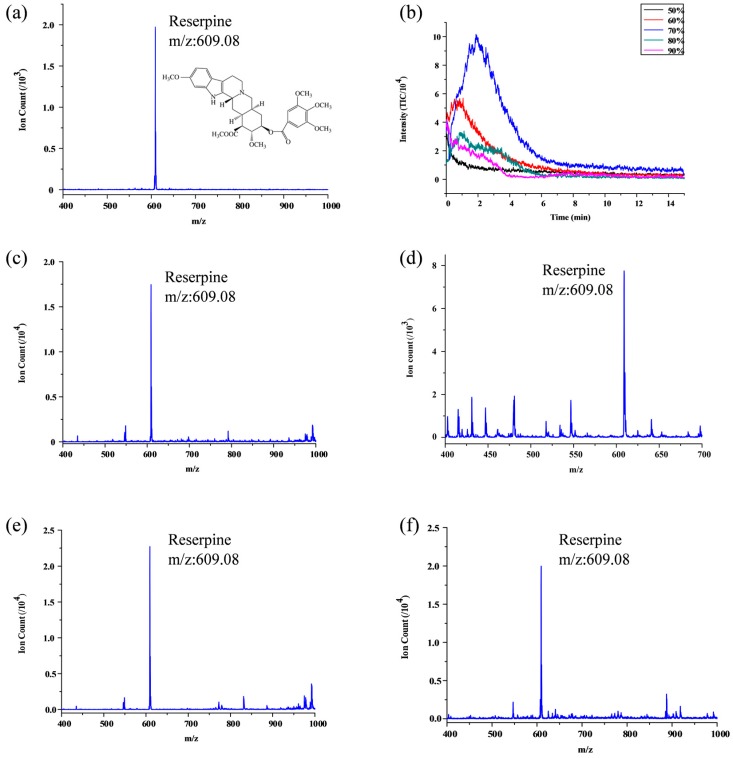
Results of SPME using standard sample: (**a**) Reserpine ion counts of the 15 min mean signal by commercial capillary; total ion current (TIC) mass range from 400 to 1000; (**b**) Different acetonitrile and water ratio with 1% formic acid used as eluent and reserpine signal change within 15 min on elution; TIC mass range from 609 to 610; (**c**) Reserpine ion counts by the FF ESI emitter that the depth of the nozzle is 260 µm; (**d**) Reserpine ion counts by the corner-integrated ESI emitter that the depth of the nozzle is 260 µm; (**e**) Reserpine ion counts by the FF ESI emitter that the depth of the nozzle is 40 µm; (**f**) Reserpine ion counts by the corner-integrated ESI emitter that the depth of the nozzle is 40 µm.

Figure 4 illustrates the extraction experiment results using standard reserpine sample with different acetonitrile and water ratio including 1% formic acid which was tested on the FF ESI emitter of 260 µm depth liquid channel for finding the optimal elution condition. The best elution condition is actually the maximum solubility of reserpine in organic solvents. It is related to solvent environment, solvent and water ratio. Figure 4b indicates that when using 70% acetonitrile with 30% water including 1% formic acid, we can acquire the best elution results around two minutes with 10^5^ ion counts (total ion current (TIC) mass range from 609 to 610); this is two order of magnitudes better than direct ESI using commercial capillary as shown in Figure 4a. Take advantage of the microfluidic with online elution and ESI without a constant volume process, we cannot estimate the enrichment factor exactly. Instead, this peak MS signal indicated the peak enrichment ability of the system. We also compare the experiment results based on the corner-integrated ESI emitter and FF ESI emitter that the depth of the liquid channel of nozzle is 260 µm under same optimal elution conditions (70% acetonitrile with 30% water including 1% formic acid) as shown in Figure 4c,d, which indicates that the signal intensity and signal-to-noise ration of the FF ESI emitter is much better than the corner-integrated ESI emitter. When liquid channel of nozzle and bottleneck have different depth, that is to say 40 µm and 260 µm respectively, the experiment results became better as shown in Figure 4e,f. According to the results, we suggested that two factors of four chip configurations affected the MS signal: firstly, the deeper open nozzle in the corner integrated ESI emitters may cause large droplets residue in the nozzle tip, which affect the MS signal; secondly, comparing the corner-integrated ESI emitter with FF ESI emitter, with nebulizer and focusing gas in FF ESI configuration, the ionizing processing is much stable and furthermore by focusing the ionizing liquid away from the PDMS way, the interferences of PDMS base can also be alleviate. As a conclusion the FF ESI with 40 µm depth nozzle illustrated the best results, we refer this as optimal chip configuration in the following illustration. However, there is a tradeoff between the effectiveness and the fabrication difficulties.

**Figure 5:**
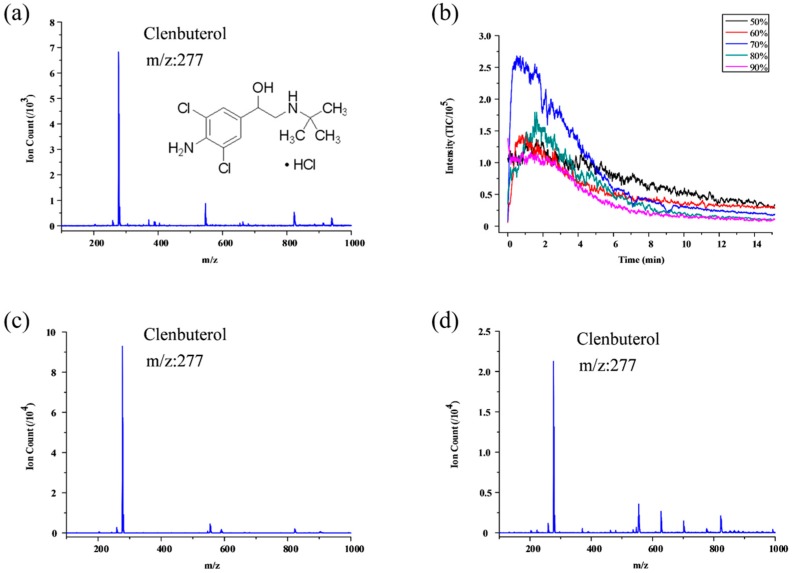
Results of SPME using standard sample: (**a**) 15.9 µM clenbuterol hydrochloride ion counts of the 15 min mean signal by commercial capillary; TIC mass range from 100 to 1000; (**b**) Different acetonitrile and water ratio with 5% ammonium hydroxide used as eluent and clenbuterol hydrochloride change within 15 min on elution; TIC mass range from 276 to 277; (**c**) clenbuterol hydrochloride ion counts by optimal chip configuration; (**d**) Clenbuterol hydrochloride ion counts by optimal chip configuration; the concentration is 3.2 µM.

**Figure 6:**
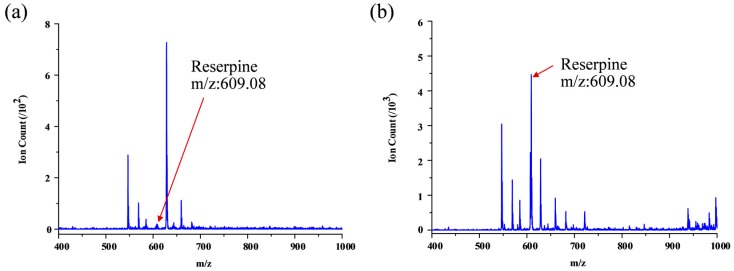
Results of SPME using urine sample spiked with 0.82 µM reserpine: (**a**) Direct ESI using a commercial capillary and (**b**) On-chip SPME and ESI using optimal chip configuration.

In addition to reserpine, we also applied SPME experiment coupling with optimal chip configuration using other analytes, such as clenbuterol hydrochloride, malachite green, sulfadiazine, etc. Figure 5 illustrates the experiment results using standard clenbuterol hydrochloride sample. Same as the reserpine experiment, different acetonitrile and water ratio including 5% ammonium hydroxide was tested for finding the optimal elution condition. Figure 5a shows 15.9 µM clenbuterol hydrochloride ion counts of the 15 min mean signal by commercial capillary. Figure 6b indicates that when using 70% acetonitrile with 30% water including 5% ammonium hydroxide, we can acquire the best elution results around two minutes with 10^5^ ion counts (TIC mass range from 276 to 277); this is also two order of magnitudes better than direct ESI using commercial capillary as shown in Figure 5a. We also did SPME experiment about the low concentration of clenbuterol hydrochloride which was about 3.2 µM and the ion counts of the 15 min mean signal by commercial capillary was about 2000. Figure 5d shows SPME experiment results that one order of magnitudes better than direct ESI using commercial capillary. 

### 3.2. Bio-Chemical Sample SPME

**Figure 7:**
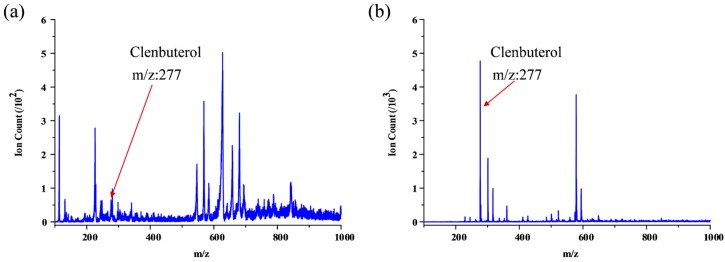
Results of SPME using urine sample spiked with 3.2 µM clenbuterol hydrochloride: (**a**) Direct ESI using a commercial capillary and (**b**) On-chip SPME and ESI using optimal chip configuration.

Furthermore, urine sample spiked with 0.82 µM reserpine was treated as real bio-chemical sample with drug metabolite. Ascribe to the high-salt and complex matrix condition for the real biological fluid, the reserpine is hard to be ionized using direct ESI; as shown in Figure 6a, very low reserpine peak can be detected under such condition; using the microfluidic SPME module coupling with integrated ESI emitters, on the other hand, we can acquire reasonable signal intensity around 5 × 10^3^ ion counts, although there still some impurity signals (as shown in Figure 6b). Same as reserpine, urine sample spiked with 3.2 µM clenbuterol hydrochloride was also applied. The clenbuterol hydrochloride was also hard to be ionized using direct ESI; as shown in Figure 7a, very low clenbuterol hydrochloride peak can be detected under such condition; using microfluidic SPME module coupling with integrated ESI emitters, we can acquire reasonable signal intensity around 5 × 10^3^ ion counts (as shown in Figure 7b). 

In addition, some literatures stated that urine has interferences that have the same mass with clenbuterol and even MS/MS transitions may lead to false positive results [27]. However, in our works, the commercial mimic urine sample we adopted is mainly constituted by urea, sodium chloride, potassium chloride, creatinine, trisodium phosphate and albumin according to the vendor data and there was no obvious suspicious components around mass range 277 according to the blank test using pure mimic urine. So we suggested that absorbed reserpine or clenbuterol hydrochloride on particles was from our spiked analytes to urine, not from urine itself. It is just a conception characterization and verification of our chip; and for further establishing a standard analytical method for real urine samples, we will adopt chromatography on-chip further or using MS^3^ transitions as in [27]. Also we will attempt to apply such chip to milk or meat samples in the future application works.

**Figure 8:**
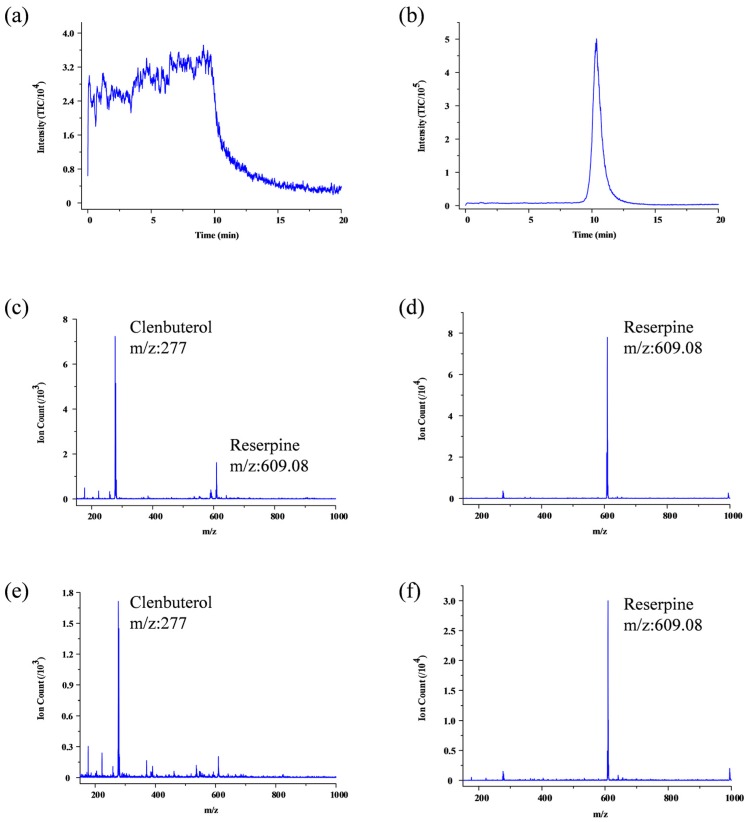
SPME experiment of reserpine and clenbuterol hydrochloride mixed solution: (**a**) clenbuterol hydrochloride (6.4 µM) change within 20 min on elution; TIC mass range from 276 to 277; (**b**) reserpine (0.205 µM) change within 20 min on elution; TIC mass range from 609 to 610; (**c**) clenbuterol hydrochloride ion counts of 0.205 µM Reserpine and 6.4 µM clenbuterol hydrochloride mixtures; (**d**) reserpine ion counts of the same mixtures as (**c**,**e**) clenbuterol hydrochloride ion counts of 0.205 µM reserpine and 3.2 µM clenbuterol hydrochloride mixtures; (**f**) reserpine ion counts of the same mixtures as (**e**).

Finally, we applied the SPME experiment of the 0.205 µM reserpine and 6.4 µM or 3.2 µM clenbuterol hydrochloride mixed solution, and realized the separation elution. In order to get a better adsorption effect for multiple analytes, we chose C18 SPME particles. Clenbuterol hydrochloride was eluted from the C18 particles firstly as shown in Figure 8a when the signal intensity was about 7000, as shown in Figure 8c. After elution completion of clenbuterol hydrochloride, reserpine signal raised (Figure 8b) which intensity was 8 × 10^4^, as shown in Figure 8d. The lowest concentration of reserpine and clenbuterol hydrochloride we used was 0.205 µM and 3.2 µM in the mixed solution with mimic biological background for illustrative results as shown in Figure 8e,f; the LOD of our system can be better than those if MS/MS transitions are utilized. However, the TIC of reserpine is much better than the TIC of the clenbuterol hydrochloride; the situation got worth if we further lower the concentration of the clenbuterol hydrochloride. This suggested a different absorption and elution ability of different analyte for single SPME conditions. Further improvement of our chip will focus on the on-chip generating different SPME environment for different analytes elution serially.

## 4. Conclusions

As a conclusion, in this paper, we have described two types of microfluidic chips (four kinds of configurations) and coupled with MS for SPME experiment. We introduce in detail the design of the chips, the fabrication processes, and the SPME particles filling process. The PDMS chips are disposable for every experiments; our previous works had shown that our PDMS emitters were quite stable during batch fabrication [11,12,13], and the integrated bottleneck structure was fully compatible with those emitters; so it did not affect the overall stability of the chip. Experiments were conducted using standard reserpine solution, standard clenbuterol hydrochloride solution and mimic urine sample to verify the on-chip SPME and MS detection functions. We compared and optimized the experiments parameters of the SPME modules with different ESI emitters. The experiment results show that this integrated module is practical, effective and application prospects is widespread. Further studies will focus on extraction and elution of multiple analytes in a single chip serially or simultaneously, and applying such chips on more complex sample matrices, such as food or biological samples in our future work.
